# Supplementary material for: Opponent visuospatial coding structures responses during memory recall and visual perception in medial parietal cortex
Source: Imaging Neurosci (Camb). 2025 Mar 24;3:imag_a_00507. doi: 10.1162/imag_a_00507 (PMC12319830; doi:10.1162/imag_a_00507)
Supplement: Supplementary Material [file imag_a_00507-supp.pdf]

Supplementary Material

Table S1: Table contains the percentage of supreahtrehsold -ve pRFs in each ROI, degrees of freedom (df), *t*-values and *p*-values for the two-tailed *t*-tests against zero.

Percentage of suprathreshold -ve pRFs					
Region	Hemisphere	% -ve pRFs	df	*t*-value	*p*-value
Places 1	LH	52.33	35	17.60	5.69^{-19}
	RH	47.87	35	22.92	1.14^{-22}
Places 2	LH	54.88	35	18.22	1.91^{-19}
	RH	49.82	35	19.56	5.78^{-22}
People 1	LH	59.90	35	23.37	6.07^{-23}
	RH	54.88	35	21.82	5.86^{-22}
People 2	LH	50.31	35	15.13	6.21^{-17}
	RH	51.73	35	18.76	7.54^{-20}

Table S2: Table contains the degrees of freedom (df), *t*-values, *p*-values and effect size estimates for the correlation between pRF timeseries RDM and Recall RDM for each ROI.

pRF timeseries RDM - Recall RDM correlation (r)					
Region	Hemisphere	df	*t*-value	*p*-value	Effect size (Cohen's d)
Places 1	LH	11	3.44	0.005	1.01
	RH	11	2.84	0.016	0.82
Places 2	LH	11	4.23	0.001	1.22
	RH	11	5.19	2.97-4	1.49
People 1	LH	11	6.35	5.40-5	1.83
	RH	11	3.29	0.007	0.95
People 2	LH	7	3.01	0.01	1.06
	RH	8	6.11	2.84-4	2.03

Table S3: Table contains the degrees of freedom (df), *t*-values, *p*-values and effect size estimates for the correlation between pRF timeseries RDM and Perception RDM for each ROI.

pRF timeseries RDM - Perception RDM correlation (r)					
Region	Hemisphere	df	*t*-value	*p*-value	Effect size (Cohen's d)
Places 1	LH	22	7.67	1.15-7	1.60
	RH	23	6.96	4.32-7	1.42
Places 2	LH	23	11.01	1.20-10	2.24
	RH	23	9.54	1.84-9	1.94
People 1	LH	23	4.89	6.08-5	0.99
	RH	23	7.26	2.14-7	1.48
People 2	LH	16	3.88	0.001	0.94
	RH	17	6.39	6.60-6	1.50
